# HELLP (Hemolysis, Elevated Liver Enzymes, and Low Platelets) Syndrome Without Hypertension Associated With Fetal Loss in the Second Trimester of Pregnancy: A Report of a Rare Case

**DOI:** 10.7759/cureus.83844

**Published:** 2025-05-10

**Authors:** Efthymia Thanasa, Anna Thanasa, Emmanouil M Xydias, Gerasimos Kontogeorgis, Ioannis Paraoulakis, Apostolos C Ziogas, Evangelos Kamaretsos, Ioannis Thanasas

**Affiliations:** 1 Department of Health Sciences, Medical School, Aristotle University of Thessaloniki, Thessaloniki, GRC; 2 Department of Obstetrics and Gynecology, EmbryoClinic IVF, Thessaloniki, GRC; 3 Department of Obstetrics and Gynecology, General Hospital of Trikala, Trikala, GRC; 4 Department of Obstetrics and Gynecology, University of Thessaly, Larissa, GRC; 5 Third Department of Obstetrics and Gynecology, University General Hospital "Attikon" Medical School, National and Kapodistrian University of Athens, Athens, GRC

**Keywords:** case report, diagnostic findings, fetal loss, hellp syndrome, management, preeclampsia, pregnancy, prognosis

## Abstract

HELLP (hemolysis, elevated liver enzymes, and low platelets) syndrome without hypertension presenting in the second trimester of pregnancy is an extremely rare obstetric complication, associated with significantly increased rates of maternal and perinatal morbidity and mortality. This case report concerns a 24-year-old multigravida woman in her 25th week of gestation, who presented to the Emergency Department of the General Hospital of Trikala, Greece, with symptoms of general malaise and clinical signs indicative of preterm labor. Following ultrasonographic confirmation of absent fetal cardiac activity, the patient was admitted to the maternity ward, where she delivered vaginally. Awaiting delivery, clinical and laboratory investigations (combined with the subsequent exclusion of other pregnancy-related pathological conditions characterized by atypical thrombocytopenia and microangiopathic hemolytic anemia) led to the definitive diagnosis of HELLP syndrome without hypertension. In this case report, we describe the rare manifestation of HELLP syndrome without hypertension during the second trimester of pregnancy, a presentation associated with fetal demise. Following the case description, a brief literature review is provided, focusing on the pathogenesis, diagnosis, and management of this rare clinical entity, whose timely and accurate treatment can significantly improve outcomes for both the mother and the fetus or neonate.

## Introduction

Hypertensive disorders of pregnancy, including chronic hypertension, gestational hypertension, and preeclampsia, are among the most common obstetric complications [1]. They are estimated to affect approximately 16% of all pregnancies and remain the leading global cause of maternal and fetal morbidity and mortality related to pregnancy [2]. Preeclampsia has been recognized since ancient times, with its first description as a distinct clinical entity dating back approximately 2000 years [3]. Affecting 2% to 8% of pregnancies worldwide, preeclampsia is a complex condition that encompasses a spectrum of hypertensive diseases in pregnancy. It can lead to end-organ damage, particularly affecting the kidneys and liver, and may progress to more severe complications, such as eclampsia and HELLP (hemolysis, elevated liver enzymes, and low platelets) syndrome [4].

HELLP syndrome is a liver disease associated with pregnancy, characterized by hemolysis, elevated liver enzymes, and low platelet count. It represents a complication or progression of severe preeclampsia and was first described in 1982 by Louis Weinstein [5]. According to some researchers, it could be considered a distinct clinical entity, as 20% of patients with HELLP syndrome do not have hypertension, and 5% to 15% of these patients present with low levels of proteinuria or no proteinuria at all [6]. HELLP syndrome is estimated to affect 0.17%-0.85% of all live births and 4%-12% of pregnancies complicated by preeclampsia. It typically presents in the third trimester of pregnancy, most commonly between the 32nd and 34th week. In up to 30% of cases, HELLP syndrome may manifest postpartum [6].

This case report describes a rare presentation of HELLP syndrome without accompanying hypertension occurring during the second trimester of pregnancy and resulting in fetal loss. Following the case presentation, a brief literature review is included, focusing on the pathogenesis, diagnostic challenges, and management strategies of this uncommon clinical entity. Appropriate and timely management of HELLP syndrome, even in its atypical forms, can significantly improve prognostic outcomes for both the mother and the fetus or neonate.

## Case presentation

A 24-year-old multigravida woman, under obstetric follow-up at a private maternity center during her current pregnancy, presented to the Emergency Department of the General Hospital of Trikala, Greece, at 25 weeks of gestation. She was in a state of general malaise and exhibited signs of preterm labor. Over the previous 48 hours, the patient reported diffuse abdominal pain, headache, nausea, vomiting, and mild vaginal bleeding. A second-trimester anomaly scan conducted ten days earlier revealed no fetal abnormalities or markers of chromosomal disorders. The patient did not provide results from a first-trimester ultrasound screening. She was a non-smoker, not overweight (BMI = 23.5), and both this pregnancy and her previous one were conceived spontaneously. Her obstetric history included a cesarean section three years earlier due to breech presentation. Her medical history was unremarkable for hepatic, renal, or cardiac conditions, and there were no autoimmune disorders such as systemic lupus erythematosus. Additionally, there was no personal history of preeclampsia or gestational diabetes in her previous pregnancy.

Upon admission, the patient’s vital signs were within normal limits: body temperature 36.8°C, blood pressure 110/70 mmHg, and heart rate 88 bpm. On physical examination, significant tenderness was noted in the epigastric region and right upper quadrant of the abdomen. Obstetric physical evaluation revealed premature uterine contractions and cervical dilation of 5 cm. Obstetric ultrasound revealed the absence of fetal cardiac activity and confirmed intrauterine fetal demise. An upper abdominal ultrasound showed a liver of normal size and echotexture, with no evidence of parenchymal lesions. Renal ultrasound was also unremarkable. The patient was transferred to the maternity ward, where she underwent laboratory evaluation (Table 1).

**Table 1:** Results of our patient's laboratory tests from admission to the maternity ward until discharge from the clinic. The presence of hemolysis, increased liver enzymes and decreased platelets < 100.000/ml and > 50.000/ml established the diagnosis of the complete HELLP (hemolysis, elevated liver enzymes, and low platelets) syndrome, category II. Ht: hematocrit; Hb: hemoglobin; PLT: platelets; WBC: white blood cells; NEUT: neutrophils; CRP: C-reactive protein; APTT: activated partial thromboplastin time; INR: International Normalized Ratio; FIB: fibrinogen; Glu: glucose; Cr: creatinine; UA: uric acid; Na+: sodium; K+: potassium; TBIL: total bilirubin; LDH: lactate dehydrogenase; SGOT: serum glutamic oxaloacetic transaminase; SGPT: serum glutamate pyruvate transaminase; AMY: amylase.

Laboratory tests	Entrance to the maternity ward	1st postpartum day	2nd postpartum day	4th postpartum day	Normal laboratory values
Ht	37.2%	36.9%	37.1%	37.4%	37.7 – 49.7%
Hb	12.7 gr/dl	12.3 gr/dl	12.4 gr/dl	12.4 gr/dl	11.8 – 17.8 gr/dl
PLT	93.5x10^3^/ml	91x10^3^/ml	97x10^3^/ml	110.5x10^3^/ml	150 – 350 x10^3^/ml
WBC	9.31x10^3^/ml	14.67x10^3^/ml	12.56x10^3^/ml	9.14x10^3^/ml	4 – 10.8 x10^3^/ml
NEUT	67.9%	81.4%	70.1%	67.3%	40 – 75%
CRP	0.37 mg/dl	0.41 mg/dl	-	-	0.5 mg/dl
APPT	28.9	27.3	-	-	24.0 – 35.0 sec
INR	0.95	0.94	-	-	0.8 – 1.2
FIB	285 mg/dl	291 mg/dl	-	-	200 – 400 mg/dl
Glu	134 mg/dl	84 mg/dl	-	-	75 – 115 mg/dl
Cr	0.48 mg/dl	0.51 mg/dl	-	-	0.40 – 1.10 mg/dl
UA	6.1 mg/dl	6.0 mg/dl	4.95 mg/dl	4.07 mg/dl	2.4 – 6.0 mg/dl
Na^+^	138.2 mmol/L	139.4 mmol/L	-	-	136 – 145 mmol/L
K^+^	4.01 mmol/L	3.87 mmol/L	-	-	3.5 – 5.1mmol/L
TBIL	1.93 mg/dl	2.01 mg/dl	1.72 mg/dl	1.43 mg/dl	0 – 1.2 mg/dl
SGOT	523 IU/L	515 IU/L	218 IU/L	101 IU/L	5 – 33 IU/L
SGPT	289 IU/L	271 IU/L	155 IU/L	65 IU/L	10 – 37 IU/L
LDH	1718 IU/L	1695 IU/L	1081 IU/L	578 IU/L	125 – 220 IU/L
AMY	73 U/mL	-	-	-	0 – 110 U/mL

While awaiting microbiological results, she was assessed by both a cardiologist and a neurologist. No signs of heart failure or pulmonary edema were observed, and neurological assessment revealed normal deep tendon reflexes and muscle tone. A random urine sample showed significant proteinuria (+++), and a 24-hour urine collection revealed a total protein excretion of 3.1 g (reference value <300 mg).

**Figure 1:**
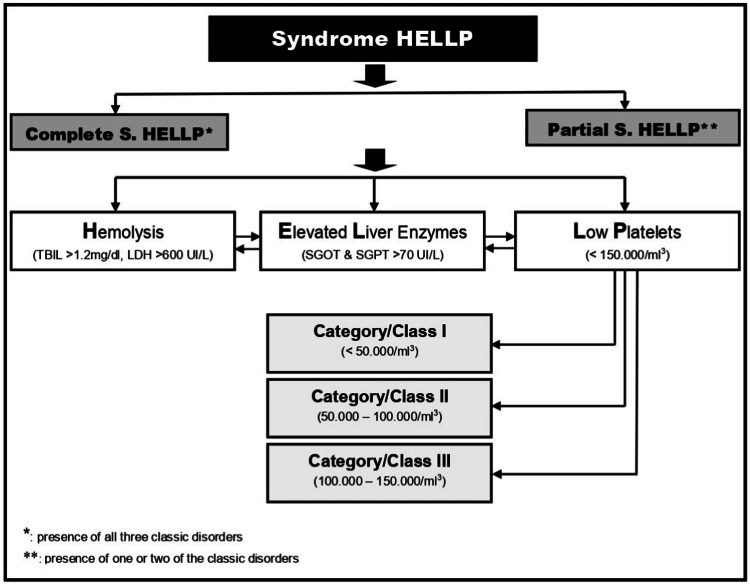
Schematic representation of HELLP syndrome classification based on the characteristic diagnostic triad (hemolysis, elevated liver enzymes, and low platelets) and total platelet count. The complete HELLP syndrome presents with all three classic disorders (as seen in our case), while partial HELLP syndrome presents with one or two of the three typical disorders. HELLP: hemolysis, elevated liver enzymes, and low platelets; S.: syndrome; TBIL: total bilirubin; LDH: lactate dehydrogenase; SGOT: serum glutamic oxaloacetic transaminase; SGPT: serum glutamate pyruvate transaminase. This image was created by the authors.

The presence of the characteristic triad-hemolysis, elevated liver enzymes, and thrombocytopenia (platelet count between 50000-100000/μL)-confirmed the diagnosis of complete HELLP syndrome without hypertension, classified as Category II (Figure 1).

Following vaginal delivery of the stillborn fetus, weighing 520 grams, placental examination revealed no signs of abruption. There was also no evidence of placental insufficiency. The patient, who remained hemodynamically stable and normotensive, was transferred to a hospital ward for continued monitoring. During hospitalization, progressive improvement in laboratory parameters was observed, with rising platelet count and declining levels of bilirubin and liver enzymes (Table 1). On the fifth day of hospitalization, the patient was discharged in good clinical condition with instructions for outpatient follow-up. Further laboratory testing excluded other pathological conditions, such as atypical thrombocytopenic disorders or microangiopathic hemolytic anemias (ADAMTS13 deficiency - thrombotic thrombocytopenic purpura, hemolytic uremic syndrome, etc.) that could have potentially complicated the pregnancy and mimicked HELLP syndrome.

## Discussion

HELLP syndrome is a multifactorial condition with an unclear pathogenesis. Several risk factors have been identified as potentially playing a key role in its development, including maternal age ≥35 years, obesity, assisted reproductive techniques, pre-existing chronic hypertension or hypertensive disorders of pregnancy, type I diabetes or gestational diabetes, chronic liver disease, chronic kidney disease, chronic cardiac conditions, and systemic lupus erythematosus [7]. Our patient presented with none of the above-mentioned risk factors. Notably, she had no history of preeclampsia during her first pregnancy and paradoxically developed HELLP syndrome during the second trimester of her second pregnancy. The occurrence of HELLP syndrome in a second pregnancy, especially in the absence of hypertension and during the second trimester, is exceedingly rare. However, the risk of recurrence in subsequent pregnancies for women with a history of HELLP syndrome or early-onset preeclampsia is significantly increased [8].

The pathogenesis of HELLP syndrome, which is closely related to that of severe preeclampsia, is primarily associated with an excessive inflammatory response in the mother, accompanied by enhanced endothelial activation [9]. The initial step in the pathogenic mechanism of HELLP syndrome is now considered to be endothelial dysfunction, which manifests as a reduced production of prostacyclin and nitric oxide. This reduction diminishes the inhibitory effect on platelet aggregation within the spiral arterioles, leading to platelet accumulation and the secretion of vasoconstrictive substances, such as thromboxane A2 and serotonin. This stimulates the renin-angiotensin system, resulting in increased production of angiotensin II, which causes vasoconstriction and raises maternal blood pressure as well as the pressure in the fetal-placental unit. This leads to endothelial dysfunction and damage. Areas of injured endothelial cells become sites for platelet and fibrin deposition, leading to thrombus formation, which, along with the hypoxia-induced by vasoconstriction, results in intravascular hemolysis and degenerative vascular damage in multiple organs [9,10].

The clinical diagnosis of HELLP syndrome presents a challenge for obstetricians, especially when it concerns pregnant women in the middle of their pregnancy who do not have hypertension (as in our case). The main clinical symptom of the disease is pain in the right upper quadrant of the abdomen or epigastrium, accompanied by nausea, vomiting, malaise, or other nonspecific symptoms that can be mistaken for an acute viral syndrome. In 20% of cases of HELLP syndrome, hypertension is absent, and 5%-15% of pregnant patients exhibit either low levels of proteinuria or no proteinuria at all [6]. In extremely rare cases, perinatal liver rupture and acute surgical abdomen due to intraperitoneal hemorrhage may occur [11]. The diagnosis of HELLP syndrome relies on laboratory findings. Hemolysis (abnormal smear indicating microangiopathic hemolytic anemia characterized by the presence of schistocytes) and hepatic dysfunction, reflected in an increase in total bilirubin > 1.2 mg/dl, an increase in lactate dehydrogenase > 600 U/l, and an increase in aminotransferases that is twice the normal limit, confirm the diagnosis of HELLP syndrome. Thrombocytopenia (platelet count <100000/ml) is the most reliable marker for diagnosing the syndrome [12,13]. Thus, HELLP syndrome, as an atypical form of thrombocytopenia and microangiopathic hemolytic anemia related to pregnancy, requires differential diagnosis from pathological conditions such as thrombotic thrombocytopenic purpura, hemolytic uremic syndrome, severe preeclampsia without HELLP syndrome, systemic lupus erythematosus, and acute fatty liver of pregnancy [14]. Furthermore, modern diagnostic methods for preeclampsia, based on placental growth factor, are believed to significantly contribute to the identification of pregnancies at the highest risk for severe complications [1].

The management of HELLP syndrome focuses on monitoring obstetric complications, controlling hypertension, preventing seizures, and determining the appropriate timing and method of delivery [15]. For the latter, it is important that delivery is expedited when possible, even in the case of known intrauterine fetal demise, in order to remove the underlying etiology of HELLP syndrome and thus ameliorate clinical manifestations. Antihypertensive treatment is indicated for the prevention of hemorrhagic stroke in cases of severe, acute-onset hypertension. However, the use of antihypertensive medications is not recommended for managing mild hypertension. For cases where HELLP syndrome leads to progressive maternal and fetal deterioration, and the risk of maternal and perinatal morbidity and mortality increases, delivery should be carried out regardless of gestational age, once the mother's condition has been stabilized [14,16]. Platelet transfusion is indicated for platelet counts <20000/ml to prevent significant hemorrhage during vaginal delivery. For cesarean sections, platelet transfusions may be required until a minimum platelet level of 50000/ml is achieved [17]. The administration of magnesium sulfate to prevent eclamptic seizures is essential in any pregnant woman with HELLP syndrome. Recent studies suggest that magnesium sulfate should also be used in women exhibiting signs of severe preeclampsia, even in the absence of neurological symptoms [18]. Prenatal administration of corticosteroids for fetal lung maturation may be considered for pregnant women with HELLP syndrome before the 34th week of gestation [19]. Finally, targeted prophylactic aspirin therapy from 12 weeks to 28 weeks of gestation in women with a high risk of preeclampsia is believed to significantly reduce the incidence of the condition in pregnancy [1].

The prognosis of HELLP syndrome is generally poor, as it is associated with severe clinical complications affecting both the mother and fetus, as well as the newborn. Disseminated intravascular coagulation, pulmonary edema, acute renal failure, liver rupture, adult respiratory distress syndrome, and cerebral hemorrhage are severe complications that can lead to maternal death. The estimated maternal mortality rate is approximately 1% of cases [20]. Perinatal mortality, primarily attributed to prematurity, placental insufficiency with or without intrauterine growth restriction, and placental abruption, is high and ranges between 7.4% and 34% [21]. The risk of recurrence of hypertensive disease in future pregnancies ranges from 27% to 48% and for HELLP syndrome in particular from 19-27% [6]. In cases where pregnancy ended before 32 weeks of gestation due to HELLP syndrome, such as the one described in this report, the risk of recurrence may be up to 61% [6]; highlighting the need for thorough and honest patient counseling and clinical vigilance during subsequent attempts at conception and pregnancy.

## Conclusions

HELLP syndrome without hypertension in the second trimester of pregnancy is an extremely rare but life-threatening condition for both the mother and the fetus. In our patient, the loss of the fetus in the second trimester of pregnancy was associated with the development of HELLP syndrome, although the exact cause leading to the intrauterine fetal death could not be precisely determined. The clinical diagnosis of HELLP syndrome poses a challenge for obstetricians, especially in cases such as the one described here, involving pregnant women in the middle of their pregnancy without hypertension. While hypertension is a common warning sign for the development of HELLP syndrome, obstetricians should remain vigilant and should not eliminate the possibility of HELLP without investigating other associated symptoms, such as right abdominal quadrant pain or tenderness as was the case in the present report, or without conducting all appropriate laboratory investigations. Early detection, accurate diagnosis, and effective management of HELLP syndrome through symptomatic management and timely labor planning, particularly expedited delivery after evaluation and stabilization of the patient, even in periviable gestational age, can contribute significantly to the reduction of maternal morbidity and mortality.

## References

[1] Wu P, Green M, Myers JE (2023). Hypertensive disorders of pregnancy. BMJ.

[2] Rosenberg EA, Seely EW (2024). Update on preeclampsia and hypertensive disorders of pregnancy. Endocrinol Metab Clin North Am.

[3] Roberts JM (2024). Preeclampsia epidemiology(ies) and pathophysiology(ies). Best Pract Res Clin Obstet Gynaecol.

[4] Allard M, Grosch S, Jouret F, Masson V, Surinder T, Masset C (2024). Prevention of preeclampsia and its complications (Article in French). Rev Med Liege.

[5] Weinstein L (1982). Syndrome of hemolysis, elevated liver enzymes, and low platelet count: a severe consequence of hypertension in pregnancy. Am J Obstet Gynecol.

[6] Rath W, Faridi A, Dudenhausen JW (2000). HELLP syndrome. J Perinat Med.

[7] Lisonkova S, Razaz N, Sabr Y (2020). Maternal risk factors and adverse birth outcomes associated with HELLP syndrome: a population-based study. BJOG.

[8] Malmström O, Morken NH (2018). HELLP syndrome, risk factors in first and second pregnancy: a population-based cohort study. Acta Obstet Gynecol Scand.

[9] Gardikioti A, Venou TM, Gavriilaki E (2022). Molecular advances in preeclampsia and HELLP syndrome. Int J Mol Sci.

[10] Abildgaard U, Heimdal K (2013). Pathogenesis of the syndrome of hemolysis, elevated liver enzymes, and low platelet count (HELLP): a review. Eur J Obstet Gynecol Reprod Biol.

[11] Larson SN, Killeen TF, Bowman L, Shankar S, Stock E, Welton L, Harmon JV Jr (2025). Hepatic rupture in HELLP syndrome: report of two patients and a review of peripartum surgical care and transfusion. Clin Case Rep.

[12] Rimaitis K, Grauslyte L, Zavackiene A, Baliuliene V, Nadisauskiene R, Macas A (2019). Diagnosis of HELLP syndrome: a 10-year survey in a perinatology centre. Int J Environ Res Public Health.

[13] Li Z, Dai Y, Yun L, Guo W (2024). A prediction model for the progression from gestational hypertension to pre-eclampsia complicated with HELLP syndrome. Int J Gynaecol Obstet.

[14] Giannubilo SR, Marzioni D, Tossetta G, Ciavattini A (2024). HELLP syndrome and differential diagnosis with other thrombotic microangiopathies in pregnancy. Diagnostics (Basel).

[15] Adorno M, Maher-Griffiths C, Grush Abadie HR (2022). HELLP syndrome. Crit Care Nurs Clin North Am.

[16] Farahi N, Oluyadi F, Dotson AB (2024). Hypertensive disorders of pregnancy. Am Fam Physician.

[17] Mol BW, Roberts CT, Thangaratinam S, Magee LA, de Groot CJ, Hofmeyr GJ (2016). Pre-eclampsia. Lancet.

[18] De Oliveira L, Korkes H, Rizzo M, Siaulys MM, Cordioli E (2024). Magnesium sulfate in preeclampsia: broad indications, not only in neurological symptoms. Pregnancy Hypertens.

[19] (2020). Gestational hypertension and preeclampsia: ACOG Practice Bulletin, Number 222. Obstet Gynecol.

[20] Erez O, Othman M, Rabinovich A, Leron E, Gotsch F, Thachil J (2022). DIC in pregnancy - pathophysiology, clinical characteristics, diagnostic scores, and treatments. J Blood Med.

[21] Osmanağaoğlu MA, Erdoğan I, Zengin U, Bozkaya H (2004). Comparison between HELLP syndrome, chronic hypertension, and superimposed preeclampsia on chronic hypertension without HELLP syndrome. J Perinat Med.

